# Prevention of Post-Transfusion Hepatitis by Screening of Antibody to Hepatitis B Core Antigen in Healthy Blood Donors

**DOI:** 10.4084/MJHID.2011.062

**Published:** 2011-12-18

**Authors:** S. Shastry, S.S. Bhat

**Affiliations:** Department of Immunohematology and Blood Transfusion, Kasturba Medical College, Manipal University, Karnataka, India

## Abstract

**Background:**

Transfusion-associated hepatitis B viral infection continues to be a major problem in India even after adoption of mandatory screening for HBsAg by ELISA method. The high incidence of TAHBV is reported in patients receiving multiple transfusions.

**Objective:**

To study the seroprevalence of hepatitis B core antibody among healthy voluntary blood donors

**Subjects and Methods:**

The study was conducted in the department of Transfusion Medicine of a tertiary care referral hospital. A total of 12,232 volunteers after passing through the stringent criteria were selected for blood donation. Donor samples were tested for all mandatory transfusion transmissible infections and anti HBc IgM (Monolisa HBc IgM PLUS:BIO-RAD, France). Reactive results were confirmed by repeat testing in duplicate. Donor data was analyzed using SPSS software and Chi-square test was used to calculate the significance of difference between the groups.

**Results:**

A total of 12,232 healthy voluntary blood donors were recruited. Majority (93.4%) were males. Median age of donor population was 26 years (range: 18–60 years). Eighty six (0.7%) were positive for HBsAg, which comes under “low prevalence (<2%) zone” as per WHO. On screening for HBcAg Ig M, 15 (0.1%) were found to be positive and none were HBsAg reactive. There was no significance of difference in the mean age between reactive and non-reactive donors.

**Conclusion:**

Evaluating the usefulness of anti-HBc screening is critical. Anti HBcAg IgM screening may be included in routine screening of donors as it is an indicator of occult HBV during window period. The cost and the unnecessary wastage of the blood units when they are positive for anti HBsAg along with the core antibody need to be studied.

## Introduction

The risk reduction strategies for transfusion transmissible infections are adopted at various stages in transfusion centers. It includes the detailed donor interview, review of the donor history, careful medical selection of the blood donors, maintenance of donor deferral register, elimination of cash payment to donors, and using of sensitive serological tests in the laboratory. Transfusion-associated hepatitis B viral infection (TAHBV) continues to be a major problem in developing countries, even after the adoption of mandatory screening for HBsAg by ELISA method. The high incidence of transfusion associated hepatitis B virus is reported in patients receiving multiple transfusion like thalassemia and hemato-oncology patients. This is mainly because of the blood from the donors with ‘occult’ HBV infection (OBI). This condition is defined as viral DNA without detectable HBsAg observed after the initial period of primary infection and most of the time accompanied by the presence of anti-HBc. Thus the absence of HBsAg in the blood of apparently healthy individuals may not be sufficient to ensure the lack of circulating HBV. and blood containing anti-hepatitis B core antibody (anti-HBc) without detectable presence of HBsAg might be infectious. However implimentation of nucleic acid testing (NAT) reduces the risk of residual infection it may not be economically feasible at every blood bank in our country. In this context we studied the seroprevalence of hepatitis B core antibody (Ig M) among blood donors at our center. We have also studied the effect of core antibody testing on discard rate of blood. This is the first study of seroprevalence of hepatitis B core antibody in South Indian blood donors.

## Subjects and Methods

The study was conducted in the department of Transfusion Medicine of a tertiary care referral hospital situated at the costal part of Karnataka State (Udupi district, India). A total of 12,232 volunteers were selected after passing through the stringent criteria for blood donation.^1^ The family members, friends or relatives of the patients were categorized as replacement donors. People who donate blood without expecting any favor in return were classified as voluntary blood donors. Donors with history of jaundice, hepatitis and high risk behavior were deferred. All the donors were counseled and informed about the tests carried out on the collected blood units. The blood samples were tested for all mandatory transfusion transmissible infections like HIV(Genscreen ULTRA, HIV Ag-Ab. BIO-RAD, France), HCV(SP NANBASE General Biologicals Corp. Taiwan), HBV(MonalisaTM HBsAg ULTRA, BIO-RAD, France), Syphilis (RPR, BIO-RAD, France) & Malaria (Qualisa Malaria, Qualpro Diagnostics, Tulip Group. Goa, India) by ELISA, Rapid Plasma Reagin testing and thick smear examinations respectively. In addition to these tests, ELISA for anti HBc IgM (Monolisa HBc IgM PLUS:BIO-RAD, France) was done. The sensitivity anti HBc IgM assay is 98.5% with analytical sensitivity of 50U/ml and specificity is 99.9% as mentioned in the product insert provided in the kit. ELISA for all the 4 markers (HIV, HCV, HBsAg, anti HBc IGM) were done simultaneously using a 4 plate automated ELISA equipment (EVOLIS by Bio RAD). Reactive results were confirmed by repeating the test in duplicate. On confirming the results, as per our departmental standard operating protocol we inform the donor confidentially. Donor demographic data was obtained and compared between reactive and non-reactive groups. Data was analyzed using SPSS software and Chi-square test was used to calculate the significance of difference between the groups.

## Results

**Figure 1:**
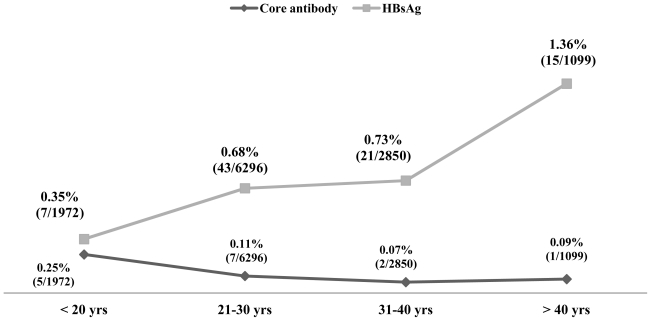
Seroprevalence of Hepatitis B Core antibody and HBsAg in different age groups of blood donors.

A total of 12232 healthy blood donors who have passed the stringent donor selection criteria were recruited for this study over a period of one year (2009–2010). Among them 8865 (72.47%) were voluntary blood donors and 3367 (27.52%) were replacement blood donors. A majority (11403, 93.2%) of them were males. Median age of donor population was 26 years (range:18–60 years). Eighty six blood donors (0.7%) were positive for HBsAg. On screening for anti-HBc (IgM), 15 (0.12%) samples gave reactive results and all of them were negative for HBsAg. Anti-HBc (IgM) reactivity was 0.03% among voluntary blood donors and, 0.35% in replacement donors (Table 1) and seroprevalence was significantly higher among replacement donors than the voluntary ones. Among 15 core antibody reactive donors only one was a female donor. Seroprevalence among male donors was 0.12% (14/11403) and among female donors was 0.11% (1/892), there was no significant difference between the two groups. On analyzing the occupation of the blood donors who were reactive for core antibody, 33% were students, 26% were businessmen and 40% as servicemen (2), electricians (2), driver (1) and barber (1). A decreasing trend of core antibody reactivity was observed with the increasing age of the donors. Seroprevalence of core IgM antibody was high in young donors with the age of <20 years and it is the lowest in donors between 31 to 40 yrs of age (Figure 1).

Sample to cut-off ratio of OD values ranged from 1.00 to 8.69 (<1.5: in 8 cases, 1.5 – 2.5: In 3 cases, > 2.5: In 3 cases). Cost analysis showed an addition of Rs.100/- per test when core antibody testing was done in addition to the other mandatory infection screening tests. Discard rate of blood was 0.88% per year due to HBV infection (HBsAg); with the addition of core antibody testing (IgM) discarding rate of increased to 0.97% per year.

## Discussion

Post transfusion hepatitis is common even after practicing the stringent donor selection criteria and screening the blood for HBsAg. This is because a considerable number of HBV infected donors remained undiagnosed, if only HBsAg is used for screening. We have studied the seroprevalence of hepatitis B core antibody among the donor population of costal Karnataka, southern part of India. Knowing the seroprevalence rate of the core antibody will provide an idea about the usefulness of the implementation of routine core antibody testing for blood donor screening purpose. In the present study we have used the kits detecting only Ig M type of antibody. The prevalence rate of core antibody is found to be 0.12% in the present study.

HBV DNA was detected in 3.3% to 30% of the blood donors those who are negative for hepatitis B surface antigen (HbsAg) and hepatitis B surface antibody (HBsAb) but positive for anti-HBc.^2^,^3^ As shown in various studies the prevalence of anti HBc antibody among blood donors varies from 0.12% to 19% (Table 2). The seroprevalence rate of anti HBc IgM in the south Indian population is not available and the present study shows a low prevalence rate in this part of country. Most of the studies from India and other countries showing high seroprevalence of core antibody have used kits with total anti HBc. A donor with a positive anti HBc-IgG indicates either a past infection or a carrier state. Anti-HBc IgG may remain positive for life in an affected individual although the individual has protective levels of anti-HBs and therefore, this does not necessarily mean that blood of such a donor is infectious. However unless we do sensitive NAT we can’t confirm the presence or absence of the virus in such donor. Unlike IgG subtype anti-HBc IgM is a marker of recent hepatitis B infection.

Our finding of significantly higher seroprevalence of core antibody among replacement donors supported the national policies aiming towards 100% voluntary blood donation. Study done by Asim et al showed a difference in the seroprevalence of core antibody between male and female donors (19.3% Vs 18%) but as in our study the difference was statistically not significant.^5^ We have observed an alarmingly high rate of core antibody positivity among younger population (with age <20 years) in contrary to the study done by Seo et al, which showed increasing prevalence of core antibody with the increasing age of the donor population.^7^ Thirty three percent of reactive donors in our study were students. Higher rate prevalence among younger population shows the need for effective implementation of preventive measures, such as prenatal testing of HBsAg in pregnant women and vaccination program. As per Indian Academy of Pediatrics, current immunization schedule for hepatitis B vaccination is, first dose at birth, second dose at 6 weeks and third dose at 6 months of age. The Hepatitis B Project was initiated in India in the year 2002 with support of Global Fund for vaccines and immunization.^14^ In addition to these measures, inclusion of educative sessions on health and hygiene may be helpful in creating awareness among younger population.

Screening of blood units for core antibody adds to the cost, but definitely useful in reducing the residual risk of post transfusion hepatitis. The false reactive results is another disadvantage of highly sensitive assays and in the present study we could not do the complete HBV panel. That is a limitation of our study. A study done in North India using IgM alone showed a little higher seroprevalence of 0.43% than the present study.^6^ However the core antibody reactivity rate was too high (15.9%) when they used kits containing both IgG and IgM. It may not be practical for any blood bank to discard such a huge amount of blood units. And in majority of blood banks in developing countries NAT may not be feasible. As mentioned by Allain et al HBV NAT is a multi-step process and each of the steps is necessary and complementary of the preceding one. Information provided by the serological markers is important to determine whether the donor is truly infected or not. The key marker at this step is anti-HBc. In an area with low HBV infection prevalence, the association of anti HBc with HBV DNA is strongly suggestive of an established infection. Whereas in high endemic areas, the value of the information is limited since a majority of the population of blood donation age carries anti HBc.^15^ Therefore, the role of anti-HBc in detecting blood donors with occult hepatitis B infection need to be considered separately in various countries.^16^ Occult hepatitis B infection has a major impact on transfusion safety. The standard test for detection of occult infection is the amplification of HBV DNA. However, the serological assay for the long lasting antibody response to the highly immunogenic HBV core antigen represents a qualified candidate as a surrogate for DNA amplification.^16^ Although HBs Ag seroprevalence rate in our study is <2%, which comes under the low prevalence area as per WHO guidelines, the implementation of core antibody testing becomes important step towards continual improvement in blood safety.

## Conclusion

Evaluating the usefulness of anti-HBc screening is critical. Anti HBcAg IgM screening may be included in routine screening of donors as it is an indicator of recent HBV infection and is the marker which is helpful in detection of HBV infection in HBsAg window period. Costal Karnataka is a low prevalence area (<2%) for hepatitis B infection. In such areas the blood discarding rate due to core antibody is not high when the kits with Ig M antibody alone is used. The cost and the unnecessary wastage of the blood units when they are positive for anti HBsAg along with the core antibody need to be studied. Analysis of cost-effectiveness will be helpful in making a policy decision for the implementation of routine screening for hepatitis B core antibody.

## Figures and Tables

**Table 1:** Hepatitis B Core antibody reactivity among different types of blood donors.

Blood donors	Anti HBc (IgM)		p-value
	Reactive	Nonreactive	
**Voluntary**	3	8862	0.000
**Replacement**	12	3355	
**Male**	14	11389	0.730
**Female**	1	828	

**Table 2:** Comparison of Seroprevalence of Anti HBc from various studies.

Place	Seroprevalence of Anti HBc	Year [Reference]
Costal Karnataka (India)	0.12%	2010 [present study]
Chandigarh (India)	8.4%	2006 [4]
New Delhi (India)	18.9%	2006 [5]
New Delhi (India)	0.43% (IgM) 15.9% (total)	2007[6]
Korea	13.5%	2008[7]
Pakistan	19.15%	2005 [8]
Iran	6.55%	2002 [9]
Italy	4.85%	2005 [10]
Turkey	15%	2006 [11]
Germany	1.52%	2002 [12]
Egypt	7.8%	2010 [13]
